# Comparison of Methods for In-House Screening of HLA-B*57:*01* to Prevent Abacavir Hypersensitivity in HIV-1 Care

**DOI:** 10.1371/journal.pone.0123525

**Published:** 2015-04-15

**Authors:** Ward De Spiegelaere, Jan Philippé, Karen Vervisch, Chris Verhofstede, Eva Malatinkova, Maja Kiselinova, Wim Trypsteen, Pawel Bonczkowski, Dirk Vogelaers, Steven Callens, Jean Ruelle, Kabamba Kabeya, Stephane De Wit, Petra Van Acker, Vicky Van Sandt, Marie-Paule Emonds, Paul Coucke, Erica Sermijn, Linos Vandekerckhove

**Affiliations:** 1 Ghent University, Department of Internal Medicine, Ghent, Belgium; 2 Ghent University, Department of Clinical Chemistry, Microbiology and Immunology, Ghent, Belgium; 3 Université Catholique de Louvain, IREC, AIDS Reference Laboratory, Brussels, Belgium; 4 Saint-Pierre University Hospital, Department of Infectious Diseases, Bruxelles, Belgium; 5 Ghent University, Center for Medical Genetics, Ghent, Belgium; 6 HILA, Laboratory for Histocompatibility & Immunogenetics Red Cross Flanders, Mechelen, Belgium; Saint Louis University Division of Infectious Diseases and Immunology, UNITED STATES

## Abstract

Abacavir is a nucleoside reverse transcriptase inhibitor used as part of combination antiretroviral therapy in HIV-1-infected patients. Because this drug can cause a hypersensitivity reaction that is correlated with the presence of the HLA-B*57:*01 *allotype, screening for the presence of HLA-B*57:*01* is recommended before abacavir initiation. Different genetic assays have been developed for HLA-B*57:*01* screening, each with specific sensitivity, turnaround time and assay costs. Here, a new real-time PCR (qPCR) based analysis is described and compared to sequence specific primer PCR with capillary electrophoresis (SSP PCR CE) on 149 patient-derived samples, using sequence specific oligonucleotide hybridization combined with high resolution SSP PCR as gold standard. In addition to these PCR based methods, a complementary approach was developed using flow cytometry with an HLA-B17 specific monoclonal antibody as a pre-screening assay to diminish the number of samples for genetic testing. All three assays had a maximum sensitivity of >99. However, differences in specificity were recorded, i.e. 84.3%, 97.2% and >99% for flow cytometry, qPCR and SSP PCR CE respectively. Our data indicate that the most specific and sensitive of the compared methods is the SSP PCR CE. Flow cytometry pre-screening can substantially decrease the number of genetic tests for HLA-B*57:*01* typing in a clinical setting.

## Introduction

Abacavir (ABC) is a nucleoside reverse transcriptase inhibitor that is used as a part of combination antiretroviral therapy in HIV-1-infected patients. In 5–8% of treated patients, ABC can induce an immune mediated hypersensitive response that correlates with the presence of the HLA-B*57:*01* allele [1–3]. Consequently, current guidelines recommend screening for the presence of the HLA-B*57:*01* allele in all HIV infected patients before ABC initiation [3].

The ABC induced hypersensitivity syndrome is accompanied by mild to moderate rash, hypotension, fever and gastrointestinal and respiratory symptoms [2]. Symptoms disappear shortly after treatment abrogation, but restart of treatment may lead to anaphylactic shock and possible death [4–6]. The strong association of the hypersensitivity response with the presence of the major histocompatibility complex (MHC) class I allele HLA-B*57:*01* was confirmed in the PREDICT-1 study (Clinical-Trials.gov ID: NCT00340080) [7–9]. Subsequently, the American case control study (SHAPE) on Caucasian and African patients confirmed a 100% correlation between HLA-B*57:*01* and a positive skin patch test combined with a hypersensitive response to ABC (Clinical-Trials.gov ID: NCT00373945) [10]. Together, these data clearly indicate the importance of HLA-B*57:*01* testing, which is now recommended before treatment initiation with ABC [3].

Numerous genotypic tests exist for HLA-B*57:*01* screening. The gold standard method, being sequence based typing of the *HLA-B* allele is laborious and expensive [11,12]. Consequently, alternative tests have been developed to increase turn-around time and decrease assay costs, while minimizing a decrease in specificity and sensitivity. A frequently used method is based on hybridization of PCR products with sequence specific oligonucleotides (SSO), followed by sequence specific primer (SSP)-PCR high resolution testing to assess whether the HLA-B*57 allele is present [13]. Currently, the most commonly used methods are based on SSP PCR [14]. This method has been further optimized by subsequent capillary electrophoresis, enhancing the sensitivity of the assay [15]. More recently, new assays were developed for HLA-B*57:*01* typing on a qPCR platform [11,16,17]. This enables detection of primer specificity through differentiating Cq values by SYBR Green quantitative (q)PCR [16], or analysis of allele specific PCR by high resolution melting [17]. Implementation of these assays on a qPCR platform significantly decreases the processing and reaction time as well as reagent costs [11].

A recent study on a 6 year long external quality assessment scheme of HLA-B*57:*01* typing in 47 laboratories from 12 different countries showed that routine clinical HLA-B*57:*01* typing using various genotypic tests resulted in a single false negative HLA-B*57:*01* report from 1283 reports, indicating that current HLA typing is excellent [18]. However, innovations in HLA typing strategies may still help laboratories decrease costs as well as turnaround time and may facilitate in-house assay development.

Apart from the PCR based methods, flow cytometry with specific monoclonal antibodies can also be used in HLA-B*57:01 screening. Although no specific antibodies for HLA-B*57:01 have been described; an antibody was recently developed that specifically binds to HLA-B*57 and HLA-B*58 [19]. Since only 10–5% of patients will test positive with this monoclonal antibody, the majority of patients will not require additional genotypic HLA-B*57:*01* testing. This strategy can substantially reduce costs, and decrease time to result in the majority of patients not requiring additional testing and sampling [20].

The present study was set up to compare different non-commercial HLA-B*57:*01* typing tests in a routine clinical setting. In the present study, two PCR based tests for genotypic HLA-B*57:*01* typing were evaluated. In addition, the complementary approach using flow cytometry to precede genotypic testing was investigated. The sensitivity, specificity and positive and negative predictive value were evaluated for their clinical utility.

## Materials and Methods

### Study design and patient samples

The present study was divided in two consecutive sub-studies (Table 1). In the first retrospective study, 41 samples were procured from patients with a known HLA-B*57:*01* positive status, as assessed by a previously validated SSO methodology (LIFECODES HLA-B SSO Typing Kit; Immucor Transplant Diagnostics) which is routinely used by the Laboratory for Histocompatibility Immunogenetics of the Belgian Red Cross. Subsequently, prospective samples from 108 patients with unknown HLA-B*57:*01* type were collected and tested using flow cytometry, genetic testing (qPCR and PCR SSP with capillary electrophoresis) and SSO as gold standard. Blood was collected in 9 ml EDTA tubes by venipuncture. For PCR, peripheral blood mononuclear cells were isolated using density gradient centrifugation with Lymphoprep (Axis Shield). Subsequently, cells were aliquoted in 1.6 ml tubes and stored as dry pellets in -80°C for qPCR until DNA isolation, performed with the GenElute blood genomic DNA kit (Sigma-Aldrich). Flow cytometry was performed on fresh blood. Fresh blood was available from all 108 patients of the blinded study, but only from 23 of the 41 patients with known HLA-B*57:*01* positive status. The study was approved by the ethical committee of Ghent University Hospital (Belgian reference number: B670201215101), this included a written consent form that was signed by the participants.

**Table 1:** Overview of the substudies with patient numbers.

	Flow cytometry	qPCR	SSP PCR CE	SSO + PCR
**Retrospective study**				
HLA-B*57:*01* +	n = 23	n = 41	n = 30	n = 41
(n = 41)				
**Prospective study**				
Blinded sampling	n = 108	n = 108	n = 96	n = 108
(n = 108)				

Representation of the substudies with the amount of samples tested per assay.

### Standard HLA-B*57:01 typing based on SSO and PCR SSP methods

Standard HLA-B*57:*01* testing was performed by a complementary SSO and high resolution SSP PCR method in a European Federation of Immunogenetics accredited Laboratory.

SSO was performed using the LIFECODES HLA-B SSO Typing Kit (Immucor Transplant Diagnostics) using the manufacturer’s recommended protocols to define the presence of the HLA-B*57. If the HLA-B*57 allele was present, the subtyping of *HLA-B57* was performed with PCR-SSP.

PCR-SSP was performed using a high resolution PCR-SSP method (Olerup SSP) using the manufacturer’s recommended protocols to discriminate between the different HLA-B*57 subtypes and to identify HLA-B*57:*01*. After validation of the SSO method for the correct identification of the HLA-B*57:*01* subtype (S1 Table) the 2^nd^ step with high resolution PCR-SSP confirmation was only performed on the HLA-B*57 positive samples if the B*57 subtype was doubtful because of inconclusive oligonucleotide hybridization results.

### HLA-B*57:01 typing by Flow cytometry

Flow cytometry was performed on a FACSCanto II instrument (BD Biosciences). 50 μL of blood was incubated with monoclonal HLA-B17 antibodies (mAb 3E12, kindly provided by BD Biosciences) labelled with phycoerythrin and CD45 labelled with FITC as previously described [19]. This antibody recognizes both HLA-B*57 and HLA-B*58 allotypes [19]. After 20 minutes incubation at room temperature erythrocytes were lysed with FACS lysis buffer and washed twice. Then samples were resuspended in 500 μL phosphate buffered saline with albumin for analysis. At least 20.000 events were acquired for analysis. Lymphocytes were gated based upon scatter and CD45 bright expression, and mean fluorescence intensity (MFI) for HLA-B17 expression was measured.

### HLA-B*57:01 typing by qPCR

The qPCR was based on the protocol described by Dello-Russo et al., that was adapted to avoid the necessity of the initial *HLA-B* PCR [16]. This assay targets the exon 2 (Primers 193F, 319R and HLA 57:01 exon 2 probe; S2 Table) and exon 3 (Primers 345F, 419R and HLAB57:01 exon 3 probe; S2 Table) region of the HLA-B*57:*01* allele. Primers were modified to increase specificity (S2 Table). An extra base was inserted at the 5’ end of primer 193F (exon 2). Three bases were removed from the 3’ end to position the primer in a more variable region of the *HLA-B* sequence. In the 345F (exon 3) primer sequence, a T to C mismatch was artificially introduced at the third position from the 3’ side. This mismatch has a limited impact on HLA-B*57:*01* amplification, but amplification is substantially impaired with non-HLA-B*57:01 amplicons that already harbor mismatches in the 3’ end region of the primer. In addition to these modifications, hydrolysis probes were used to increase specificity. These probes contain a 5’ FAM fluorophore, a 3’ black hole quencher combined with an internal ZEN quencher (Integrated DNA Technologies).

QPCR was performed on a LightCycler 480 system (Roche Applied Sciences) using the LightCycler 480 Probes master mix (Roche Life Sciences). Each reaction was performed in duplicate in 5 μl reaction mix containing 1 μl (50–150 ng) of DNA isolate, 500 nM forward and reverse primers and 10 nM of the hydrolysis probe. Cycling conditions consisted of an initial denaturation of 5 min at 95 °C, then 40 cycles of a denaturation step at 95°C for 15 sec, and an annealing/elongation step at 61°C. After cycling, quantification cycles (Cq) of each reaction were obtained using the second derivative method within the Lightcycler 480 software (Roche Life Sciences, version: SW 1.5).

Raw Cq values that differed by more than 8 cycles between one of the three reactions, i.e. exon 1, exon 2 and *RPP30* (primers RnasePf, RnasePR, RnaseP probe; S2 Table) were considered as HLA-B*57:*01* negative. Samples with smaller differences in Cq values were labeled positive for HLA-B*57:*01*.

This modified qPCR method was compared to the assay originally described by Dello Russo et al., in HLA-B*57:*01* positive patients [16]. For this assay a first PCR was performed to amplify a 922 bp fragment of the *HLA-B* allele using primer pair 5BIn1-57 and 5BIn3-37 (S2 Table). PCR reactions were performed in 20 μl containing 2 μl of 10x PCR buffer (Life Technologies), 120 μM dNTPs, 2 mM MgCL2, 0.4 U Platinum Taq polymerase (Life Technologies), 300 nM primers and isolated DNA at a final concentration of 1 ng/μl. PCR cycling consisted of a first polymerase activation step at 95°C for 10 min, followed by 35 times denaturation at 95°C for 20 seconds and an annealing/extension step at 68°C for 1 min in an Applied Biosystems 2720 thermal cycler (Life Technologies). The PCR product was run on the LabChip GX system (PerkinElmer) for microfluidic electrophoresis to assess whether the *HLA-B* amplicon was properly amplified. Subsequently, qPCR was performed using the previously described primers (S2 Table) [16]. PCR was performed in 5 μl using the 5X LightCycler 480 Probes mastermix (Roche Life Sciences), with 0.25 μl of 20x LightCycler 480 ResoLight Dye (Roche Life Sciences), 500 nM of the primers and the product of the first PCR in a 1:10, 1:100 and 1:1000 dilution.

### HLA-B*57:01 typing by Sequence specific PCR with capillary electrophoresis (SSP PCR CE)

SSP PCR CE was performed as previously described using sequence specific primers (S2 Table) [14,15]. The PCR reaction was performed in a total volume of 20 μl and consisting of 3–10 ng input DNA, 1.25 μM primer 1F, 0.5 μM primers 2R, 3R and 4R and 0.25 μM primers HGH-F and HGH-R (S2 Table), KAPA PCR buffer to a final concentration of 1s, 1.5 mM MgCl_2_, 0,025 U KAPA Taq Hot Start polymerase (KAPA Biosystems) and 0.12 mM dNTPs (Life Technologies). Cycling conditions included an incubation step for 3 min at 95°C followed by 30 cycles of 1 min at 95°C, 1 min at 63°C and 1 min at 72°C and a final incubation of 10 min at 72°C in an Applied Biosystems 2720 thermal cycler (Life Technologies).

The PCR products were run on an Applied Biosystems 3130xL automated capillary sequencer (Life Technologies) with GeneScan 500 ROX dye size standard (Life Technologies) and formamide for fluorescent readout of PCR fragments. Data analysis was performed using the GeneMapper software (Life Technologies, S1 Fig).

### Statistical analysis

Standard non parametric statistical analysis was performed using the wilcox.test function in the R based MASS package (version 7.3–35).

## Results

### Retrospective analysis of samples with known HLA-B*57 status (Table 2)

Because of the low frequency of the HLA-B*57 allele in the general population, an initial retrospective study on samples with known HLA-B*57 status was performed to allow a more accurate estimation of the sensitivity of the assays.

**Table 2:** Characteristics of the tested methods on the retrospective samples.

		Flow cytometry			qPCR			SSP PCR CE		
SSO + SSP PCR	N	HLA-B17	non HLA-B17	NA	HLA-B*57:*01*	non-HLA-B*57:01	NA	HLA-B*57:*01*	non-HLA-B*57:01	
HLA-B*57:01	41	23	0	18	41	0	0	32	0	9
non-HLA-B*57:01	0	0	0		0	0	0	0	0	
total	41	23		18	41		0	32		9
Sensitivity (%)		>99			>99			>99		

Characteristics of the three tested HLA-typing methods on the retrospective samples compared to the SSO and high resolution SSP PCR as gold standard. (NA: Not Available).

Flow cytometry screening with the monoclonal HLA-B17 antibody using an original cut-off at an MFI of 500 revealed a bright expression with an average MFI of 1.14x10^4^ (SD = 4.35x10^3^) in all 23 retrospectively collected samples known to be positive for HLA-B*57:*01* (S3 Table), indicating a >99% sensitivity of the flow cytometry screening (Table 2).

The qPCR method was positive for all 41 HLA-B*57:*01* confirmed samples indicating a >99% sensitivity of the qPCR based screening (Table 2). Average Cq differences in positive samples between exon 2 and *RPP30* PCRs was: 1.49 (SD = 0.713); between exon 3 and *RPP30* PCRs was: 2.12 (SD = 0.87); and between exon 2 and exon 3 PCRs was: 0.71 (SD = 0.53).

SSP PCR CE was performed on 30 confirmed HLA-B*57:*01* positive samples. All samples were correctly typed as HLA-B*57 positive indicating a >99% sensitivity for the SSP PCR CE method (Table 2).

### Prospective analysis of samples with unknown HLA-B*57 status (Table 3)

#### Flow Cytometry

Flow cytometry screening was performed on 108 prospectively collected samples with unknown HLA-B*57:*01* status. This resulted in positive flow cytometry results for 17 samples (16%) of which only 2 patients were determined as HLA-B*57:*01* positive by SSO (S4 Table). Of the 91 samples that were negative by flow cytometry, all samples were confirmed negative by SSO. Within the 17 HLA-B17 positive samples, a comparison of the MFI of all HLA-B*57:*01* positive samples versus HLA-B*57:*01* negative revealed that the latter had a significantly lower MFI (p<0.0001 by Wilcoxon rank sum test) (S2 Fig). Assessment of the HLA alleles that resulted in positive flow cytometry signals with the HLA-B17 antibody was subsequently performed by high resolution SSP PCR (Table 3 and S4 Table). This revealed that only 3 samples with the HLA-B*57:*03* allotype and 2 samples with the HLA-B*58 allotype resulted in fluorescent signals that were indiscernible from the fluorescent signals of HLA-B*57:*01* positive samples (S4 Table). Based on this data, the samples were re-assessed using a less stringent cut-off at an MFI of 5000.

**Table 3:** Characteristics of the tested methods on the prospective samples.

			Flow cytometry			qPCR			SSP PCR CE		
SSO + SSP PCR	N	HLA-B17		non HLA-B17	NA	HLA-B*57:*01*	non HLA-B*57:*01*	NA	HLA-B*57:*01*	non HLA-B*57:*01*	NA
HLA-B*57:*01*	2		2	0		2	0		2	0	0
non HLA-B*57:*01*	106		15	91		3	103		0	92	14
total	108		17	91	0	5	103		2	92	14
PPV			11.8			40.0			100.0		
NPV			100.0			100.0			100.0		
Sensitivity (%)			100.0			100.0			100.0		
Specificity (%)			85.8			97.2			100.0		
TAT			same day			1 day			1–2 days		

Characteristics of the three tested HLA-typing methods compared to the SSO and high resolution SSP PCR as gold standard. The data from the flow cytometry study is based on the most stringent cut-off at an MFI of 500. (NA: Not Available; PPV: Positive Predictive Value; NPV: Negative Predictive Value; TAT: Turn Around Time).

With the original cut-off at an MFI of 500, flow cytometry reached a sensitivity of >99%, a specificity of 85.8%, a positive predictive value (PPV) of 11.8% and a negative predictive value (NPV) of >99%. A re-analysis of the samples using the new, less stringent cut-off at an MFI of 5000 increased the PPV of 11.8% (using the initial cut-off at an MFI of 500) to 28.6% and maintained the NPV at >99%.

#### qPCR

QPCR was performed on 108 prospectively collected samples. Analysis of the samples with unknown HLA-B*57:*01* status revealed that the two HLA-B*57:*01* positive samples were detected by qPCR. In addition to these, three false positive samples were detected by qPCR compared to SSO. High resolution PCR SSP revealed that these samples all contained the HLA-B*57:*03* allele. These results indicate a sensitivity of >99% and specificity of 97.2%, a PPV of 40% and an NPV of 100% (Table 3).

#### Sequence specific PCR with capillary electrophoresis (SSP PCR CE)

SSP PCR CE was performed on 96 of the 108 unknown samples from the blinded study. This method gave positive results for all confirmed positive samples.

From the unknown samples, the two HLA-B*57:*01* were detected as HLA-B*57:*01* positive and the HLA-B*57:*03* samples were assessed as HLA-B*57 positive but different to HLA-B*57:*01*. These results indicate that both the assay sensitivity and specificity had a value of >99% in the currently investigated samples (Table 3).

## Discussion

The present paper describes the comparison of three different methods for HLA-B*57:*01* typing that are suitable for use in a routine clinical laboratory, either as stand-alone tests or in a stepwise protocol. The current data indicate that all tested assays had a maximal sensitivity, as no patients were falsely allocated as HLA-B*57:*01* negative.

The high sensitivity of the flow cytometry assay with the HLA-B-17 monoclonal antibody at detecting HLA-B*57:*01* positive samples renders this method a preferred assay for upfront screening. However, the relatively low specificity of flow cytometry compared to genetic tests makes it unsuitable as a stand-alone tool. Using flow cytometry 14% of patients will test false positive for HLA-B*57:*01* as measured in the present cohort. Implementation of multiple monoclonal HLA-B*57:*01* antibodies may increase the specificity of a flow cytometry test [21]. However, genetic screening remains necessary for samples with positive results using the HLA-B17 antibodies. It should be noted that the limited turn-around time of the flow cytometry allows fast decision making. Additionally, flow cytometry may have a high value in resource limited settings where flow cytometry is often more accessible and more affordable compared to genetic screening tools [22].

The flow cytometry analysis of samples with unknown HLA type indicated that the HLA-B17 antibody detects other alleles than previously reported [19,20]. There was a lower fluorescence in many of these false positive samples compared to the true HLA-B*57:*01* positive samples (S2 Fig), but a stringent cut-off has to be applied to avoid the majority of false negatives. In the present cohort, a possible cut-off set at an MFI of 5000 increased the PPV of 11.8% (with the original cut-off at an MFI of 500) to 28.6% while the NPV at >99% was maintained.

The qPCR based screening allows an expedited screening of the HLA-B*57:*01* type compared to the currently described qPCR assays. Contrarily to the assay described by Dello-Russo et al, the present assay is not performed as a nested PCR [16]. Preliminary data with the nested PCR design, indicated that the first PCR of the nested PCR protocol did not always result in amplification of the *HLA-B* amplicon. The amplification efficiency of the first PCR is crucial as a suboptimal PCR may render a positive sample false negative. Consequently, the direct qPCR strategy with hydrolysis probes was preferred. An additional advantage of the direct PCR consists of the internal reference gene (here *RPP30*) used to normalize for variations in the sample input, and to assess sample quality, further enhancing the flexibility of the assay.

Despite the advantages of the qPCR assay, HLA-B*57:*03* alleles were indiscernible from HLA-B*57:*01* positive samples (data not shown). This contrasts to the results observed earlier using the nested PCR approach [16]. However, also with the nested set-up we were unable to observe a difference between the B*57:03 and B*57:01 subtypes. This finding indicates that the qPCR methodology has a lower specificity as previously reported, making it suboptimal compared to the SSP PCR with capillary electrophoresis. Consequently, the latter method should be considered as a preferred method over the qPCR if capillary electrophoresis equipment is available.

In conclusion, our data indicate that the most specific and sensitive of the compared tests among those compared are the SSP PCR CE and the SSO. A combined approach with pre-screening by flow cytometry forms a valid alternative to limit the amount of genotypic testing.

## Supporting Information

S1 Fig Flow cytometry based HLA-B17 typing. *A & B*: Flow cytometry fluorescence intensity histograms of lymphocytes stained with the HLA-B17 antibody for an HLA-B*57:*01* positive (A) and negative sample (B), showing a clear discrimination of HLA-B17 positive versus negative samples. **C:** Scatterplot of Mean Fluorescence Intensities (MFI) of HLA-B17 positive samples that were either HLA-B*57:*01* positive or negative based on SSO and SSP PCR as the gold standard. Apart from the HLA-B*58 (cubes) and HLA-B*57:*03* (diamonds), all other non- HLA-B*57:*01* alleles (triangles) had a markedly lower MFI with the HLA-B17 antibody compared to the HLA-B*57:*01* positive samples (dots).
(TIF)

S2 Fig SSP PCR CE based HLA-B*57:*01* typing. Electropherograms of the SSP PCR CE assay with specific bin for HLA-B*57:*01 (left)*, HLA-B*57 (middle) and for the positive control (right). Sample A is HLA-B*57:*01* positive, as it results in a positive peak in each bin. Sample B contains the HLA-B*57:*03* allele (as assessed by high resolution PCR SSP) and has a positive signal in the middle bin, but not in the left bin. Sample C is HLA-B*57 negative, as it has no positive peak in the left and middle bin, but a clearly positive peak in the right bin, showing that the reaction was succesful, but that the HLA-B*57:*01* allele was not present.
(TIF)

S1 Table Validation of the PCR SSO. Overview of the validation data of PCR-SSO correlated to the PCR-SSP high resolution results as previously performed with a set of confirmed HLA-B*57:*01* positive samples. Oligonucleotides in the SSO typing kit that react with HLA-B*57:*01* to HLA-B*57:*04* (n = 14) are indicated in blue, oligonucleotides that define the differences between HLA-B*57:*01* to HLA-B*57:*04* (n = 8) are indicated in red and oligonucleotides that react with specificities on other HLA alleles are indicated in black and are not relevant for this study.
(XLSX)

S2 Table Primers and probes. Primers and probes used in this study, including the specific annealing temperatures (Ta) for the qPCR.
(XLSX)

S3 Table Individual test results of the retrospective study. Test results of each patient for the retrospective study, including the gold standard method (SSO combined with high resolution SSP PCR), qPCR, SSP PCR CE and flow cytometry.
(XLSX)

S4 Table Test results of the prospective study. Test results of each patient in the prospective study, including the gold standard method (SSO combined with High Resolution PCR), qPCR, SSP PCR CE and flow cytometry.
(XLSX)
