# Supplementary figures and images for: Comparison of Methods for In-House Screening of HLA-B*57:01 to Prevent Abacavir Hypersensitivity in HIV-1 Care
Source: PLoS One. 2015 Apr 15;10(4):e0123525. doi: 10.1371/journal.pone.0123525 (PMC4398410; doi:10.1371/journal.pone.0123525)

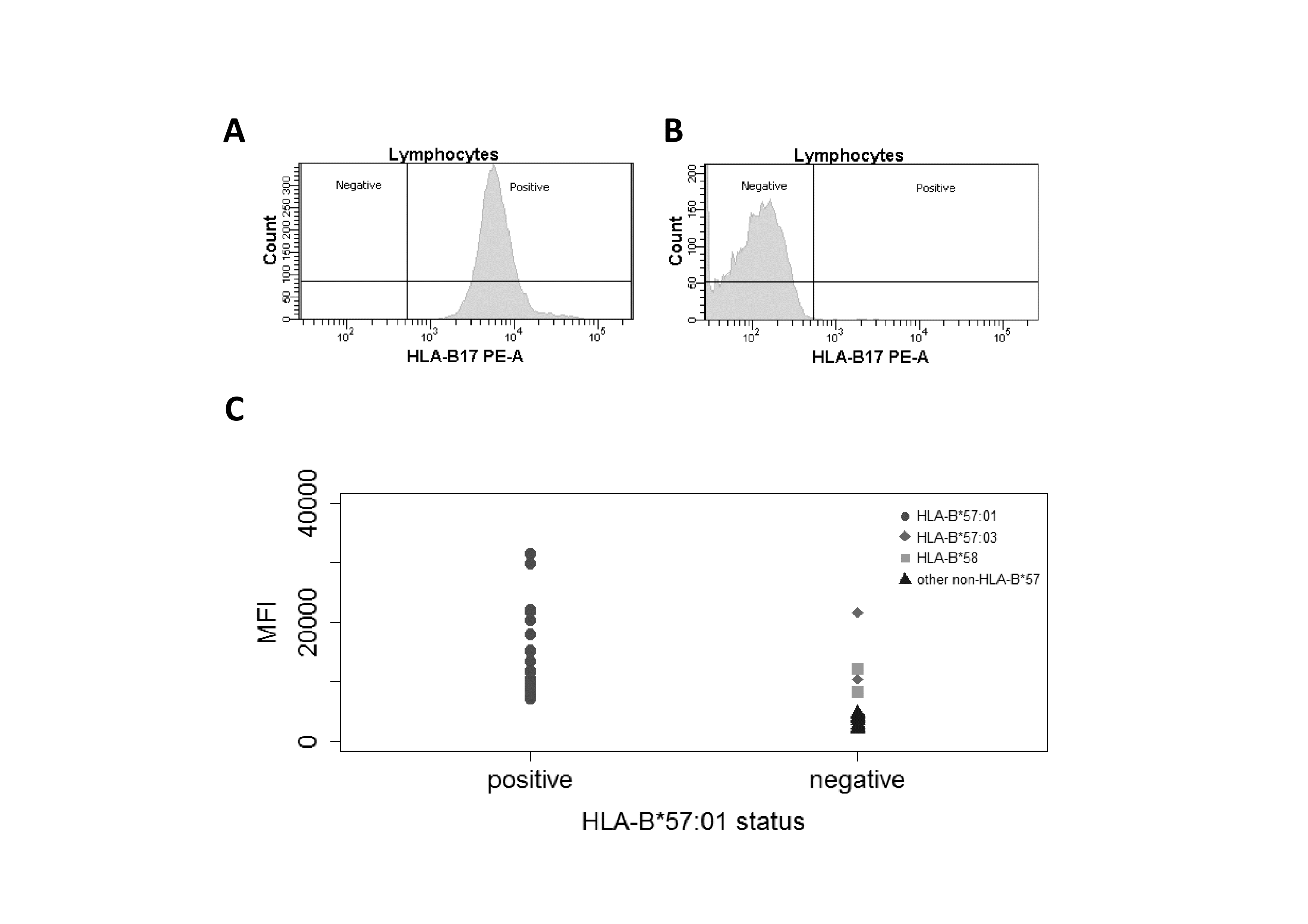

Supplement: S1 Fig — A & B: Flow cytometry fluorescence intensity histograms of lymphocytes stained with the HLA-B17 antibody for an HLA-B*57:01 positive (A) and negative sample (B), showing a clear discrimination of HLA-B17 positive versus negative samples. C: Scatterplot of Mean Fluorescence Intensities (MFI) of HLA-B17 positive samples that were either HLA-B*57:01 positive or negative based on SSO and SSP PCR as the gold standard. Apart from the HLA-B*58 (cubes) and HLA-B*57:03 (diamonds), all other non- HLA-B*57:01 alleles (triangles) had a markedly lower MFI with the HLA-B17 antibody compared to the HLA-B*57:01 positive samples (dots). (TIF) [file pone.0123525.s001.tif]

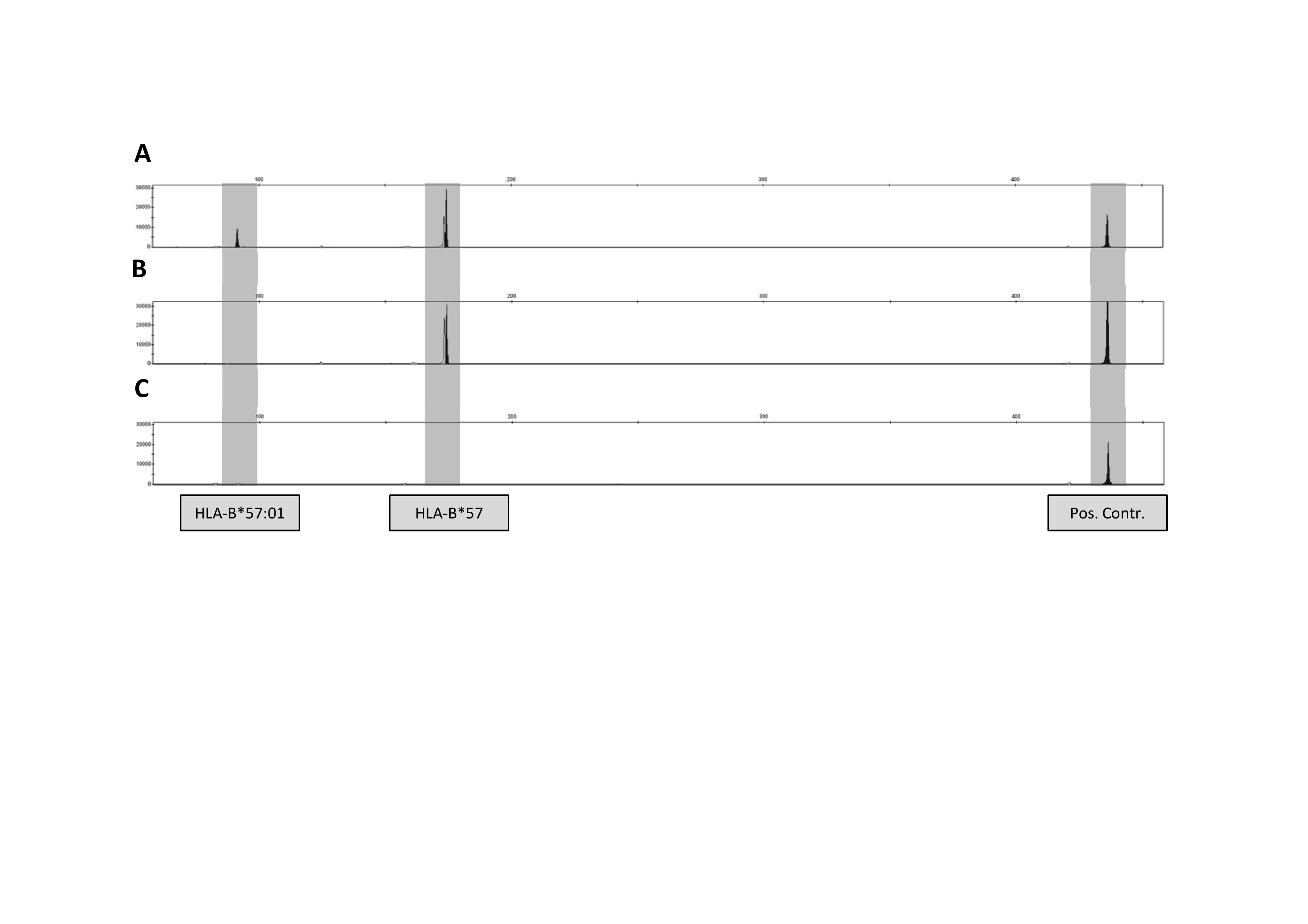

Supplement: S2 Fig — Electropherograms of the SSP PCR CE assay with specific bin for HLA-B*57:01 (left), HLA-B*57 (middle) and for the positive control (right). Sample A is HLA-B*57:01 positive, as it results in a positive peak in each bin. Sample B contains the HLA-B*57:03 allele (as assessed by high resolution PCR SSP) and has a positive signal in the middle bin, but not in the left bin. Sample C is HLA-B*57 negative, as it has no positive peak in the left and middle bin, but a clearly positive peak in the right bin, showing that the reaction was succesful, but that the HLA-B*57:01 allele was not present. (TIF) [file pone.0123525.s002.tif]
